# Social Media Interventions to Promote HIV Testing, Linkage, Adherence, and Retention: Systematic Review and Meta-Analysis

**DOI:** 10.2196/jmir.7997

**Published:** 2017-11-24

**Authors:** Bolin Cao, Somya Gupta, Jiangtao Wang, Lisa B Hightow-Weidman, Kathryn E Muessig, Weiming Tang, Stephen Pan, Razia Pendse, Joseph D Tucker

**Affiliations:** ^1^ School of Media and Communication Shenzhen University Shenzhen China; ^2^ University of North Carolina Project-China Guangzhou China; ^3^ SESH Global Guangzhou China; ^4^ World Health Organization South-East Asia Region New Delhi India; ^5^ School of Journalism and Communication Jinan University Guangzhou China; ^6^ School of Medicine University of North Carolina at Chapel Hill Chapel Hill, NC United States; ^7^ Gillings School of Global Public Health University of North Carolina at Chapel Hill Chapel Hill, NC United States

**Keywords:** social media, HIV, MSM, intervention, testing, adherence

## Abstract

**Background:**

Social media is increasingly used to deliver HIV interventions for key populations worldwide. However, little is known about the specific uses and effects of social media on human immunodeficiency virus (HIV) interventions.

**Objective:**

This systematic review examines the effectiveness of social media interventions to promote HIV testing, linkage, adherence, and retention among key populations.

**Methods:**

We used the Preferred Reporting Items for Systematic Reviews and Meta-Analyses (PRISMA) checklist and Cochrane guidelines for this review and registered it on the International Prospective Register of Systematic Reviews, PROSPERO. We systematically searched six databases and three conference websites using search terms related to HIV, social media, and key populations. We included studies where (1) the intervention was created or implemented on social media platforms, (2) study population included men who have sex with men (MSM), transgender individuals, people who inject drugs (PWID), and/or sex workers, and (3) outcomes included promoting HIV testing, linkage, adherence, and/or retention. Meta-analyses were conducted by Review Manager, version 5.3. Pooled relative risk (RR) and 95% confidence intervals were calculated by random-effects models.

**Results:**

Among 981 manuscripts identified, 26 studies met the inclusion criteria. We found 18 studies from high-income countries, 8 in middle-income countries, and 0 in low-income countries. Eight were randomized controlled trials, and 18 were observational studies. All studies (n=26) included MSM; five studies also included transgender individuals. The focus of 21 studies was HIV testing, four on HIV testing and linkage to care, and one on antiretroviral therapy adherence. Social media interventions were used to do the following: build online interactive communities to encourage HIV testing/adherence (10 studies), provide HIV testing services (9 studies), disseminate HIV information (9 studies), and develop intervention materials (1 study). Of the studies providing HIV self-testing, 16% of participants requested HIV testing kits from social media platforms. Existing social media platforms such as Facebook (n=15) and the gay dating app Grindr (n=10) were used most frequently. Data from four studies show that HIV testing uptake increased after social media interventions (n=1283, RR 1.50, 95% CI 1.28-1.76). In the studies where social media interventions were participatory, HIV testing uptake was higher in the intervention arm than the comparison arm (n=1023, RR 1.64, 95% CI 1.19-2.26).

**Conclusions:**

Social media interventions are effective in promoting HIV testing among MSM in many settings. Social media interventions to improve HIV services beyond HIV testing in low- and middle-income countries and among other key populations need to be considered.

**Trial Registration:**

International Prospective Register of Systematic Reviews (PROSPERO): CRD42016048073; http://www.crd.york.ac.uk/PROSPERO/display_record.php?ID=CRD42016048073 (Archived by WebCite at http://www. webcitation.org/6usLCJK3v)

## Introduction

Since 2000, global efforts at human immunodeficiency virus (HIV) control have had significant effects. Acquired immune deficiency syndrome (AIDS)-related deaths have been reduced [1], and 19.5 million people are accessing antiretroviral therapy (ART) [2]. To achieve the end of the AIDS epidemic by 2030, 90% of all people living with HIV (PLWH) should know their HIV status, 90% of all people with diagnosed HIV infections should receive ART, and 90% of all people receiving ART should have viral suppression by 2020 [3]. However, in 2016, only 70% of PLWH globally had been diagnosed, 53% were receiving ART, and 44% had achieved viral suppression [4]. Improved efforts are needed to reach PLWH with a comprehensive package of HIV interventions, including HIV testing, linkage to care, ART, and retention. More programs are needed for key populations who are disproportionately affected by HIV and who have difficulty accessing services across the HIV care continuum [5]. The World Health Organization defines key populations as men who have sex with men (MSM), people who inject drugs (PWID), prisoners, sex workers, and transgender people [5,6]. Barriers to HIV interventions among key populations are complex and include persistent stigma and discrimination, punitive laws, and low risk perception [7-9]. Innovative approaches to reach these populations with equitable, accessible, and acceptable services will be essential to achieve 90-90-90 targets [3].

As social media has expanded globally, these platforms have been adopted to deliver HIV interventions, especially for key populations [10,11]. Social media is defined as an Internet-based platform that allows the creation and exchange of user-generated content, usually using either mobile or Web-based technologies [12]. Popular social media platforms, such as Facebook and YouTube, have over 1.5 billion monthly active users as of June 2017 [13]. Social media possesses the characteristics of interactivity, allows users to generate content [14], and attracts high user engagement [15]. Specifically, social media can enable convenient access, at any time and place, to information and services on stigmatized diseases such as HIV. In addition, social media can be used to form online communities to seek social support, which is known to improve treatment adherence and uptake of HIV services [16].

Systematic reviews have been conducted recently on the relationship between social media and HIV outcomes [10,11,17,18]. These studies generally define social media broadly (eg, to include eHealth and mHealth interventions) or examine usage of social media for a variety of purposes, including recruitment, surveillance, communication, and HIV prevention and treatment. These studies have not focused on social media interventions among key populations [5,6]. Key populations are of special importance because they influence epidemic dynamics and likely have a disproportionate influence on the effectiveness of the response to HIV [5]. To address these gaps in a rapidly growing field, this systematic review and meta-analysis looks at the effectiveness of social media interventions in promoting HIV testing, linkage to care, adherence to treatment, and retention along the HIV care continuum among key populations.

## Methods

### Conduct of Systematic Review

This systematic review was conducted following the Preferred Reporting Items for Systematic Reviews and Meta-Analyses (PRISMA) guidelines [19]. In August 2016, the following databases were systematically searched without restriction on publication date: PubMed, Cochrane Library, Cumulative Index to Nursing and Allied Health Literature (CINAHL), Embase, Scopus, and Sociological Abstracts. Three conference databases (Conference on Retroviruses and Opportunistic Infections; International AIDS Society Conference on HIV Science, and Youth+Tech+Health Conference) were also searched for abstracts for 2015 and 2016.

### Search Strategy and Selection Criteria

The search strategy was designed with a librarian to identify studies regarding social media interventions to promote HIV testing, linkage, adherence, and retention among key populations (Multimedia Appendix 1). It was developed based on key terms, medical subject headings (MeSH) terms, synonyms, and subject headings related to three groups: (1) HIV, (2) social media or social media category or social media platform, including the most popular social networks (websites and apps) and top gay dating apps worldwide selected on the basis of their popularity and number of active users [20-22], and (3) key populations, including MSM, PWID, sex workers, and transgender persons.

Following the PRISMA guidelines [19], key study characteristics such as population, intervention, and outcomes defined the eligibility criteria. In particular, studies were included when (1) study population included key populations such as MSM, PWID, sex workers, and transgender individuals, (2) the intervention was created or implemented on at least one social media platform (existing platform or new platform with social networking components), and (3) outcomes included promoting HIV testing, linkage to care and treatment, adherence to ART, or retention. The inclusion criterion did not have any restrictions on geography or setting, except the language of publication as English. Studies without a comparator arm were also included. Studies in which social media were used for marketing/advertising, surveillance, recruitment, or data collection purposes only were excluded. Commentary, protocol, featured article, published articles without full text or sufficient details on interventions or outcomes, and modeling studies were also excluded.

Two investigators independently reviewed all abstracts identified through searches and screened them for eligibility. The full texts of the abstracts that met the eligibility criteria were then reviewed to confirm inclusion in the analysis. Disagreements were resolved through discussion with a third reviewer.

### Data Extraction

Data extraction for the included studies was completed using a standardized extraction form in Microsoft Excel that included the following information: first author, study design (randomized controlled trial [RCT] or observational study), study date, study location, sample size, target population (MSM, PWID, transgender individuals, and/or sex worker), intervention dates/duration, social media platforms, the role of social media, the reach of social media intervention, whether there were interactions in-person or offline events, step in the HIV cascade, and study outcomes. The “role of social media” was categorized into (1) social media used to develop intervention materials for promoting HIV services, (2) social media used to establish virtual peer-mentored or online communities that promote HIV interventions, (3) social media as a platform to offer HIV-related services, such as HIV self-testing kits order and request, and (4) social media as a platform to disseminate HIV-related information [23]. Among various social media interventions, those who used interactive characteristics, tailored contents, or peer influence of social media were considered as participatory social media interventions. For studies with a comparator arm, data were abstracted on the type of interventions, participants, and outcomes for both the intervention and the comparator arm.

### Quality Assessment

Two reviewers assessed the quality of the included studies using the checklist tool in Sanderson et al [24], and a third reviewer collated the results. For each study, the following six domains were used to assess risk of bias: (1) methods for selecting study participants, (2) methods for measuring exposure and outcome variables, (3) design-specific source of bias, (4) method of control confounding, (5) statistical methods, and (6) other biases (including conflict of interest and disclosure of funding sources). The Cochrane Collaboration’s recommendations [25] were used to categorize each of the six domains as “low risk of bias” (“+”) or “high risk of bias” (“–”).

### Statistical Analysis

For RCTs meeting the inclusion criteria, pooled relative risks (RR) were used to compare the participants in the intervention and the comparator arm with respect to HIV testing rates among total participants. Pooled RR were also used to compare HIV testing outcomes at baseline and post social media interventions. Data from studies on HIV self-sampling were pooled to summarize (1) the proportion of total participants requesting HIV self-sampling services, (2) the proportion of participants requesting HIV self-sampling who returned their test kits, and (3) HIV positivity rates. Meta-analyses were conducted by Review Manager, version 5.3. Pooled RR and 95% confidence intervals (CI) were calculated using random-effects models.

## Results

### Study Characteristics

**Figure 1:**
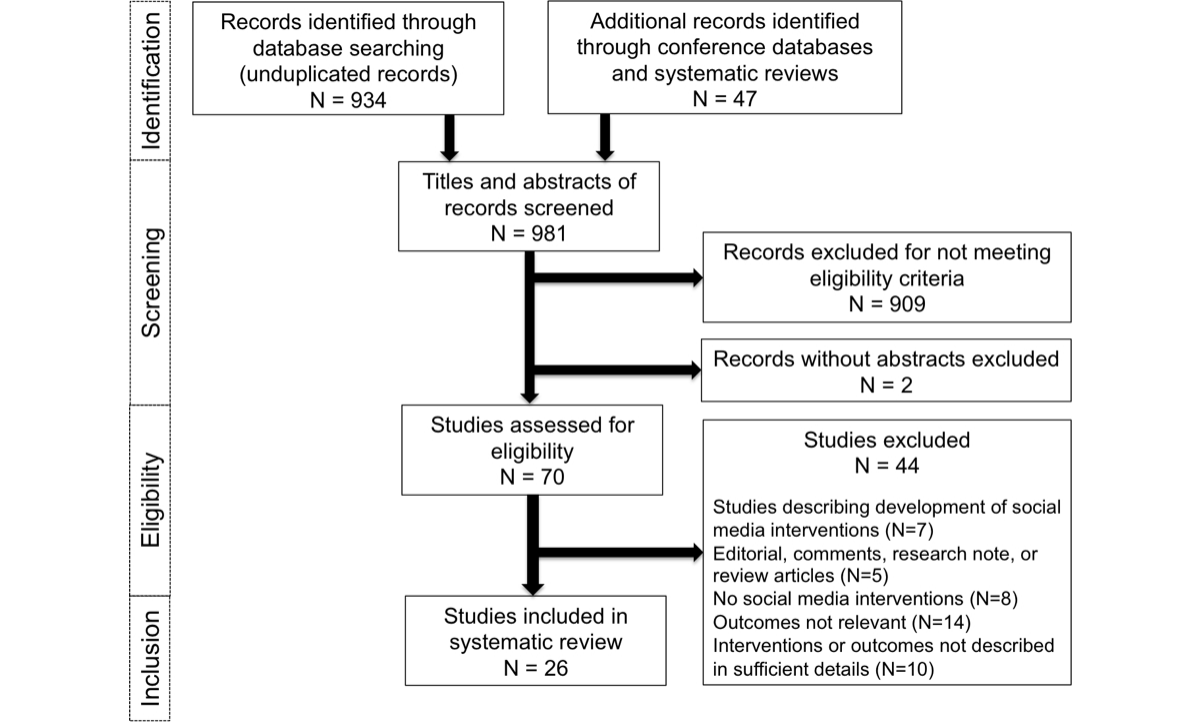
PRISMA chart showing study selection process.

Of the 981 articles and abstracts, 26 studies met our inclusion criteria [26-51] (Figure 1). Of the 26 studies, eight were RCTs [28,33,41,42,46,47,49,50] and 18 were observational studies [26,27,29-32,34-40,43-45,48,51] (Table 1). These studies were implemented in 10 countries from 2007-2015 and published from 2011-2016. In total, 18 studies were from four high-income countries as defined by the World Bank [28,29,31-38,40,42-45,47,48,50], 10 studies were from the United States [28,33-35,38,42,43,45,47,50], five from the United Kingdom [29,31,32,36,48], two each from Australia [40,44], China [46,51], and Thailand [26,27], and one each from Taiwan [37], India [41], Peru [49], Mexico [30], and Guatemala [39]. The reach of social media interventions varied from 55 to over 17,000 individuals.

### Key Populations

MSM were the primary population included in the reviewed social media interventions. All of the included studies contained MSM populations, and five also covered transgender individuals [26,27,35,42,46]. In three studies, MSM with the following specific characteristics were included: young MSM [28], young black MSM [47], and MSM living with HIV [33].

### Social Media Platforms

Existing social and sexual networking sites and gay-specific websites/apps were used for interventions. Fifteen studies used Facebook [26-29,32,33,35-37,39,41,44,47,49,50] and 10 studies used Grindr, a social networking app catering to MSM [29,32,34-36,38-40,45,48]. Other generic social networking platforms included YouTube, Twitter, and QQ, and other social networking sites or apps catering to MSM included Gaydar, Jack’d, Scruff, A4A, and Radar. Six studies used both social and sexual networking sites as intervention platforms [29,32,35,36,39,51]. Five studies created their own social media platform to provide HIV services [26-28,30,33].

### Role of Social Media

In one study, social media was used as a crowdsourcing tool to develop intervention materials (video) for promotion of HIV testing [46]. The study showed that the crowdsourced video arm (114/307, 37.1%) had similar results as the social marketing arm (111/317, 35.0%) in promoting HIV tests, but the cost of crowdsourced intervention was less than the social marketing intervention per first-time HIV test (US $131 vs US $238 per person) and per new HIV diagnosis (US$415 vs US $799 per person).

Ten studies reported using social media to establish virtual communities where HIV services (testing and adherence) were promoted [26,33,37,40,42,43,45,47,49,50]. While two studies built their own platforms to form interactive communities [26,33], the remaining studies established these communities on Facebook, popular gay dating apps or existing chat rooms [37,40,42,43,45,47,49,50]. Interventions provided in the communities included online HIV counseling, educating people about HIV and importance of HIV testing, referral for HIV testing, and answering questions related to HIV. HIV testing uptake was higher in virtual communities that emphasized peer-to-peer interaction among participants [37,49].

Social media also served as a platform for delivering services such as HIV self-testing kits, HIV self-sampling, or home-based HIV testing (9 studies) [27,29,31,32,34,36,44,48,50]. MSM were often recruited through a broadcast message as a pop-up or a personal message or promotional banner on social media, and the most popular platforms were Facebook and Grindr [29,32,34]. Finally, HIV-related information was disseminated using social media in nine studies [28-30,35,38,39,41,44,51]. Some studies tailored the content to be distributed on social media by user’s race/ethnicity and age based on quantitative data and evaluated through community advisory boards and youth advisory boards [28]. Other studies trained outreach health educators on using gay-specific apps to provide MSM with HIV education, counseling, and testing information [38,39].

In three studies, social media played more than one role [29,44,50]. In 14 studies, the online activities that were a part of the social media interventions were augmented with offline events or in-person interaction [26,27,29-32,35,36,38,39,44,48,49,51]. For example, during or post social media interventions, HIV testing were provided at clinics or home [27,36,38,51] and in studies on HIV test home-sampling, test results were conveyed to participants over the phone or via messages [29].

**Table 1:** Characteristics of the studies included in the systematic review (n=26).

Study	Study design	Study location	Sample size	Target population	Intervention period/ duration	Social media platforms	Role of social media	Interaction in-person or offline	Step in the HIV cascade
Anand 2015 [26]	Observational	Thailand	11,120	MSM & transgender individuals	2011.09-2014.12 (ongoing)	Facebook, Line, “Adam’s Love” online platform	Build community^a^	Yes	HIV testing and linkage to care
Anand 2016 [27]	Observational	Thailand	97	MSM & transgender individuals	2015.12-2016.05	Facebook, Line, “Adam’s Love” online platform	Deliver kits^b^	Yes	HIV testing
Bauermeister 2015 [28]	RCT	US	130	Young MSM	30 days	Facebook; “Get connected” platform	Disseminate^c^	No	HIV testing
Brady 2014 [29]	Observational	UK	17,629	MSM	Phase 1: 2013.01- 2013.09; Phase 2: 2013.11- 2014.03	Grindr, Gaydar, Manhunt, Facebook, Twitter	Deliver kits (blood) & disseminate	Yes	HIV testing and linkage to care
Buzdugan 2016 [30]	Observational	Mexico	61	MSM	5 weeks	Online game app	Disseminate	Yes	HIV testing
Elliot 2012 [31]	Observational	UK	321	MSM	2011.11.07- 2012.01.11	Gaydar	Deliver kits	Yes	HIV testing
Elliot 2016 [32]	Observational	UK	17,361	MSM	2012-2014	Gaydar, Grindr, Facebook	Deliver kits (oral)	Yes	HIV testing and linkage to care
Horvath 2013 [33]	RCT	US	123	MSM living with HIV	2011.02-2011.04	Facebook; “Thrive with me” platform	Build community	No	ART adherence
Huang 2015 [34]	Observational	US	16,328 (112 in survey)	MSM	Phase 1: 2014.04.17- 2014.05.29; Phase 2: 2014.10.13- 2014.11.11	Grindr	Deliver kits (oral)	No	HIV testing
Hyden 2016 [35]	Observational study	US	274	MSM & transgender individuals	2013.03-2017.12 (ongoing)	Facebook, Scruff, Grindr, Online game App	Disseminate	Yes	HIV testing
Jones 2015 [36]	Observational	UK	305	MSM	2014.11.29- 2014.11.30 (2 days)	Facebook, Grindr, Squirt	Deliver kits (blood)	Yes	HIV testing
Ko 2013 [37]	Observational	Taiwan	1037	MSM	2011.04-2011.09	Facebook	Build community	No	HIV testing
Lampkin 2016 [38]	Observational	US	903	MSM	2012.10-2013.03; 2013.10-2014.03	Grindr	Disseminate	Yes	HIV testing
Mendizabal-Burastero 2016 [39]	Observational	Guatemala	7244	MSM	2014.07-2015.12	Facebook, Twitter, Grindr, WhatsApp	Disseminate	Yes	HIV testing and linkage to care
Munro 2016 [40]	Observational	Australia		MSM	Not mentioned	Grindr	Build community	No	HIV testing
Patel 2016 [41]	RCT	India	244	MSM	2015.02-2015.05	Facebook, WhatsApp	Disseminate	No	HIV testing
Rhodes 2016 [42]	RCT	US	1292	MSM & transgender individuals	2013.07-2014.06	Adam4Adam, BlackGayChat, Craigslist, Gay.com	Build community	No	HIV testing
Rhodes 2011 [43]	Observational	US	509	MSM	2009.02-2009.07	Online chat room	Build community	No	HIV testing
Roberts 2015 [44]	Observational	Australia	503	MSM	2013.11 & 2014.07	Facebook, YouTube, Twitter	Deliver kits & disseminate	Yes	HIV testing
Sun 2015 [45]	Observational	US	2709	MSM	2013.08-2014.02	A4A Radar, Grindr, Jack’d, Scruff	Build community	No	HIV testing
Tang 2016 [46]	RCT	China	721	MSM & transgender individuals	2014.09	MSM dating websites	Develop materials^d^	No	HIV testing
Washington 2016 [47]	RCT	US	142	Young black MSM	Not mentioned	Facebook	Build community	No	HIV testing
West 2015 [48]	Observational	UK	55	MSM	2014.11 (5 days)	Grindr	Deliver kits	Yes	HIV testing
Young 2015 [49]	RCT	Peru	556	MSM	Phase 1: 2012.03.19- 2012.06.11; Phase 2: 2012.09.26- 2012.12.19	Facebook	Build community	Yes	HIV testing
Young 2013 [50]	RCT	US	112	MSM	2010.09-2011.01	Facebook	Build community & deliver kits	No	HIV testing
Zou 2013 [51]	Observational	China	429	MSM	2007.06-2007.08	Gay dating & volunteer websites, QQ, online gay chatroom	Disseminate	Yes	HIV testing

^a^“Build community” is when social media is used to establish virtual peer-mentored or online communities that promote HIV interventions.

^b^“Deliver kits” is when social media serves as a platform to deliver newly designed or extant evidence-based HIV intervention.

^c^“Disseminate” is when social media is used as a platform to disseminate HIV-related information.

^d^“Develop materials” is when social media is used to develop intervention materials for promoting HIV services.

### Uptake of HIV Testing and HIV Home Sampling

**Figure 2:**
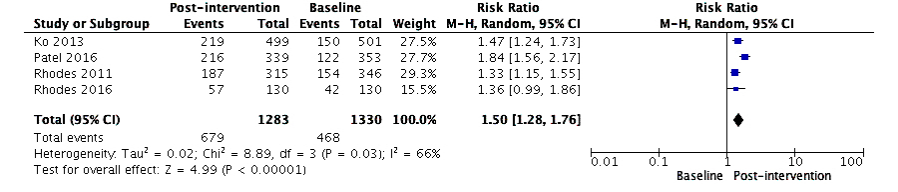
Comparison between studies with baseline and postintervention data on HIV testing (n=4).

We found 25 studies that used social media interventions to promote HIV testing [26-32,34-51] among MSM or MSM and transgender individuals, while one study used it for improving ART adherence [33]. Of these 25 studies, seven were RCTs [27,40,41,45,46,48,49] and the remaining 18 were observational studies [26,27,29-32,34-40,43-45,48,51]. Four studies were pooled to compare the rate of HIV testing at baseline and postintervention [37,41-43]. The meta-analysis showed that the HIV testing rate significantly increased after the social media interventions were provided (RR=1.50, 95% CI 1.28-1.76, *I*^2^ 66%; Figure 2).

**Figure 3:**
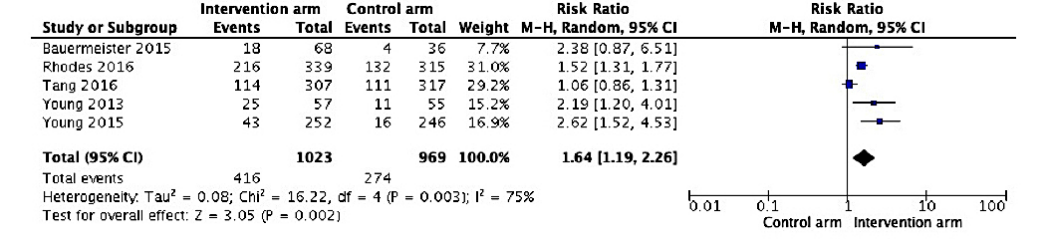
Comparison of HIV testing uptake between the intervention arm and the comparison arm in the RCTs (n=5).

Data from five RCTs show that when social media interventions were participatory [28,42,46,49,50], HIV testing rates were significantly higher (RR 1.64, 95% CI 1.19-2.26, *I*^2^ 75%; Figure 3) than in the comparative arm, where there were no social media interventions, or the social media interventions provided general health information or were not participatory. Of the seven RCTs, Patel et al [41] was excluded since both arms were interventional arms while Washington et al [47] was excluded from the analysis due to nonavailability of complete data. Similar to included studies, in Washington et al [47], those in the intervention arm (HIV testing video + chat) were seven times more likely to test for HIV than those in control group receiving standard HIV text information (study not included in pooled RR due to incomplete data).

Four studies in the United Kingdom and United States used social media to offer HIV self-testing services to MSM [29,31,32,50] (Tables 2,3, and 4). On average, 15.65% of the participants requested HIV testing kits (n=67,054; 95% CI 15.37-15.92), ranging from 15.50% to 36.36% (data from three studies) [30,31,49]. Nearly 57% of the participants who requested test kits returned them (n=24,703; 95% CI 55.92-57.16). On average, the HIV positivity rate was 1.51% among those who received results (n=13,956; 95% CI 1.32-1.73) (data from three studies [29,31,32,34]).

### Quality Assessment

There was a high risk of bias in methods for selecting study participants in 15 studies [27,29-32,34-36,38-40,42-44,48] and in measuring exposure and outcome bias in 13 studies [26,29,31,34-36,38-40,44,45,48,51]. Design-specific source of bias was present in 10 studies [27,28,30,36,39,41,45,46,49,50]. Concerns regarding control confounding bias were in 14 studies [26,27,29-32,34,35,37,40,44,45,48,51] and concerns of statistical methods in 15 studies [26,29-32,34-36,38-40,44,48,49,51] (Multimedia Appendix 2). Only two studies had low risk of bias in all the 6 dimensions [33,47]. See Multimedia Appendix 3 for results of the included studies.

**Table 2:** Studies that use social media to provide HIV self-testing services (n=3): Request for HIV test kits.

Study	Total participants/webpage views	Request testing kit	Request percentage (95% CI)
Elliot 2012 [31]	363	132	36.36 (31.58-41.43)
Elliot 2016 [32]	66,579	10,323	15.50 (15.23-15.78)
Young 2013 [50]	112	36	32.14 (24.21-41.26)
Total	67,054	10,491	15.65 (15.37-15.92)

**Table 3:** Studies that use social media to provide HIV self-testing services (n=4): Return rate of test kits.

Study	Request for testing kit	Returned testing kit	Return percentage (95% CI)
Brady 2014 [29]	14,212	8187	57.61 (56.79-58.42)
Elliot 2012 [31]	132	73	55.30 (46.79-63.52)
Elliot 2016 [32]	10,323	5696	55.18 (54.22-56.13)
Young 2013 [50]	36	11	30.56 (18.00-46.86)
Total	24,703	13,967	56.54 (55.92-57.16)

**Table 4:** Studies that use social media to provide HIV self-testing services (n=4): HIV positivity rate.

Study	Total participants tested	HIV positive	Positive rate (95% CI)
Brady 2014 [29]	8187	111	1.36 (1.13-1.63)
Elliot 2012 [31]	73	4	5.48 (2.15-13.26)
Elliot 2016 [32]	5696	96	1.69 (1.38-2.05)
Total	13,956	211	1.51 (1.32-1.73)

## Discussion

### Principal Findings

Social media have played varied roles and have been used in multiple settings to promote public health interventions. For example, social media was found to be effective in promoting physical activity [52] and smoking cessation [53]. Previous reviews have also found that social media interventions can improve HIV-related outcomes such as promoting HIV testing and linkage to care [10,11,17,18]. This systematic review shows that social media interventions are being used to promote HIV testing among MSM and transgender populations. In the studies included, HIV testing uptake among this key population group increased after implementation of social media interventions. Also, HIV testing uptake was particularly higher when the social media interventions were participatory and peer-driven. These findings are consistent with other studies [54,55] and support implementation of social media interventions that reach MSM populations and promote MSM participation.

HIV testing is the main focus of social media interventions along the HIV care continuum and is critical for achieving the first Joint United Nations Programme on HIV/AIDS (UNAIDS) 90-90-90 target [3]. Four studies found that HIV self-testing kits distributed through social media led to large numbers of requests for and return of test kits. World Health Organization guidelines strongly recommend HIV self-testing as an additional approach to HIV testing services [56]. One study on HIV self-testing included in this review concludes that providing self-testing through social media is acceptable and cost-effective [34]. Cost per person tested was US $39 and US $26 of this cost was attributed to test kits [57]. This is comparable to other research that found self-testing (US $9.23) costs lower than facility-based testing (US $11.84) in Malawi [58]. The current studies that use social media to reach hard-to-reach populations and offer HIV self-sampling or self-testing services were implemented in high-income countries where social media penetration rates are high. More usability research on HIV self-testing using social media is needed in low- and middle-income countries. Meanwhile, all included articles reported using social media to promote HIV testing, except that one study aimed to enhance ART adherence [33]. In addition to promoting HIV testing, future research should explore how social media can be leveraged to promote other HIV care services.

Social media interventions that were participatory and peer-led resulted in higher HIV testing rates compared with those that had no social media interventions or social media interventions that did not include interactive characteristics. Engaging people to participate in the interventions can increase their perceived relevance and importance of the activities [59]. Participatory strategies of social media interventions, such as tailoring intervention contents to user’s characteristics [28,49] and encouraging peer interactions [49,50] and community contributions [46], can increase the likelihood for participants to link to HIV care and services. Provided that participatory and peer-led social media interventions allow the organizers to receive feedback from the users and can motivate users to take actions, future social media interventions should adopt established strategies or develop new strategies to improve engagement and interaction with participants.

Among the studies included in this review, a variety of social media platforms were used for promoting HIV services. They included existing online social and sexual networking platforms and standalone HIV-focused websites or apps. Over half of the studies used Facebook or one of the globally or nationally popular gay dating apps. Existing platforms may be useful for HIV interventions as they often have wide reach, high user engagement and retention, and attract specific key populations that would otherwise be difficult to recruit [60]. On the other hand, five of the 26 studies developed standalone platforms. Such platforms could be used to design more tailored and innovative interventions, but using only standalone platforms may have the drawback of poor retention and limited coverage [61]. Few studies used other popular social media platforms such as Twitter and YouTube, partly because such platforms are often used as knowledge, news, or video sharing platforms but not to develop long-term relationships for continuous service provision [62]. Research is needed to assess the appropriateness and effectiveness of these platforms to deliver HIV-specific interventions for key populations.

All studies in this review primarily focused on MSM populations. This may be due to the early adoption and popularity of social and sexual networking sites among this population. Online sex seeking sites and apps for MSM provide an important channel for seeking social support and meeting new sexual partners [63,64]. While use of these platforms has been associated with increased risk behaviors for HIV [65,66], they have also been effectively used for promotion of HIV testing [47,67]. Although several studies included transgender people in addition to MSM, results were not disaggregated and studies were not powered to evaluate differences in transgender populations. The feasibility and acceptability of social media interventions and the appropriate social media platforms to deliver HIV services within networks of PWID and sex workers is also underresearched. Given disparities along the prevention and care continuum with these populations [68] and strong within-group networks [69], the potential of using social media in these populations should be actively explored.

### Limitations

This systematic review found that studies on social media intervention have several limitations. First, the current research’s methodological scoring was low due to the fact that the majority of the studies were exploratory and descriptive. Of the 26 studies, 24 had high or unclear risk of bias for at least one of the bias items in the methodological quality assessment. The high risk of bias indicates relatively low quality of studies on social media interventions, hindering more pooled analysis and causing possible bias of this meta-analysis. Second, there was also a variety of control groups used in the seven RCTs on HIV testing. For example, in the control groups, either no interventions were provided or social media was used to provide general health-related information or promote HIV services without interactive features and peer-leaders. Third, some studies used apps to improve linkage and retention in care, but for one-way monitoring of patients instead of bidirectional interactions.

This systematic review also has its own limitations. First, it was restricted to English language publications and was not able to cover studies in other languages. However, an empirical analysis found that this was not associated with bias [70]. Second, due to the heterogeneity of intervention types and measures in outcomes, we had difficulties pooling certain data. Some pooled results were largely driven by a few studies. This meta-analysis was not able to compare effects of social media intervention on MSM versus transgender individuals and through standalone platform versus existing platforms. Last, currently many studies on this topic are underway, so the state-of-the-science will continue to grow rapidly over the next few years.

### Future Studies

This systematic review shows the effectiveness of using social media interventions to improve HIV testing among MSM population and has implications for both future research and public policy. Gaps should be filled on using social media to promote HIV services beyond HIV testing and among key populations beyond MSM. Moreover, how to maximize the use of social media to promote HIV service needs further exploration. In addition, given that social media interventions have been found to be effective, there is an opportunity for national programs to leverage social media to support scale-up of such interventions. In particular, low- and middle-income countries, where mobile usage is rising, seem to have relatively few social media interventions.

### Conclusion

A total of 26 articles were identified in this systematic review to examine the role of rapidly expanding social media in improving access to HIV-related interventions and its effect in promoting HIV services among key populations. Social media can contribute to creating innovative intervention programs, disseminating intervention information, building virtual communities, and especially promoting HIV self-testing and self-sampling. Social media interventions were effective in increasing HIV testing rates, especially in high-income countries, aimed at MSM. To achieve the goal of 90-90-90 by 2020 and ultimately end AIDS by 2030, adapting these social media interventions in low- to middle-income countries and other key populations may be useful.

## Appendix

Multimedia Appendix 1 Search terms.

Multimedia Appendix 2 Quality assessment of the included studies.

Multimedia Appendix 3 Results of included studies.

## Abbreviations

AIDS: acquired immune deficiency syndrome
ART: antiretroviral therapy
CINAHL: Cumulative Index to Nursing and Allied Health Literature
HIV: human immunodeficiency virus
MSM: men who have sex with men
PLWH: people living with HIV
PRISMA: Preferred Reporting Items for Systematic Reviews and Meta-Analyses
PWID: people who inject drugs
RCT: randomized controlled trial
RR: relative risk
UNAIDS: The Joint United Nations Programme on HIV/AIDS
UNC: University of North Carolina
