# Supplementary material for: Social Media Interventions to Promote HIV Testing, Linkage, Adherence, and Retention: Systematic Review and Meta-Analysis
Source: J Med Internet Res. 2017 Nov 24;19(11):e394. doi: 10.2196/jmir.7997 (PMC5722976; doi:10.2196/jmir.7997)
Supplement: Multimedia Appendix 1 [file jmir_v19i11e394_app1.pdf]

Table S1: Search terms

No.	Terms
1	HIV OR human immunodeficiency virus OR AIDS OR acquired immunodeficiency syndrome OR acquired immune deficiency syndrome OR acquired immuno-deficiency syndrome
2	social media OR blogging OR webcast OR web 2.0 OR medicine 2.0 OR health 2.0 OR blog OR podcast OR Facebook OR web log OR YouTube OR twitter OR tweet OR gay apps OR social web OR social network OR social networking OR geosocial networking OR mobile app OR mobile application OR web2 OR social software OR social medium OR social gaming OR microblogging OR vodcast OR Google Plus OR Google+ OR Instagram OR LinkedIn OR Pinterest OR Reddit OR Second Life OR Tumblr OR Weibo OR WeChat OR Grindr OR Jack'd OR Scruff OR Growlr OR OkCupid OR QQ OR Qzone OR Skype OR Snapchat OR V Kontakte OR Whatsapp OR Baidu tieba OR Viber OR Zank OR Hornet OR Messenger OR Blued
3	bisexual OR bisexuality OR bisexuals OR gay OR GLB OR GLBT OR homosexual OR homosexuality OR homosexuals OR intersex OR LGB OR LGBT OR LGBTQ OR men who have sex with men OR MSM OR queer OR OR sexual minority OR sexual orientation OR transgender OR transgendered OR transsexual OR transsexualism OR transsexualism OR transsexuality OR sex worker OR FSW OR female sex worker OR PWID OR people who inject drugs OR people who use drugs OR injecting drug users OR IDU OR drug user OR substance users OR prisoner OR prisoners OR incarcerate OR inmate OR key population OR high risk population
4	(#1 AND #2 AND #3)
