# Supplementary material for: Social Media Interventions to Promote HIV Testing, Linkage, Adherence, and Retention: Systematic Review and Meta-Analysis
Source: J Med Internet Res. 2017 Nov 24;19(11):e394. doi: 10.2196/jmir.7997 (PMC5722976; doi:10.2196/jmir.7997)
Supplement: Multimedia Appendix 2 [file jmir_v19i11e394_app2.pdf]

Table S2: Quality assessment of the studies included

Study	Methods for selecting study participants	Methods for measuring exposure and outcome variables	Design-specific source of bias	Method of control confounding	Statistical methods	Other bias
Anand 2015	+	–	+	–	–	+
Anand 2016	–	+	–	–	+	+
Bauermeister 2015	+	+	–	+	+	+
Brady 2014	–	–	+	–	–	+
Buzdugan 2016	–	+	–	–	–	+
Elliot 2012	–	–	+	–	–	+
Elliot 2016	–	+	+	–	–	+
Horvath 2013	+	+	+	+	+	+
Huang 2015	–	–	+	–	–	+
Hyden 2016	–	–	+	–	–	+
Jones 2015	–	–	–	+	–	+
Ko 2013	+	+	+	–	+	+
Lampkin 2016	–	–	+	+	–	+
Mendizabal-Burastero 2016	–	–	–	+	–	+
Munro 2016	–	–	+	–	–	+
Patel 2016	+	+	–	+	+	+
Rhodes 2011	–	+	+	+	+	+
Rhodes 2016	–	+	+	+	+	+
Roberts 2015	–	–	+	–	–	+
Sun 2015	+	–	–	–	+	+
Tang 2016	+	+	–	+	+	+
Washington 2016	+	+	+	+	+	+
West 2015	–	–	+	–	–	+
Young 2013	+	+	–	+	–	+
Young 2015	+	+	–	+	+	+
Zou 2013	+	–	+	–	–	+

Note: (+) means low risk of bias and (–) means high risk of bias.
