# Supplementary material for: Social Media Interventions to Promote HIV Testing, Linkage, Adherence, and Retention: Systematic Review and Meta-Analysis
Source: J Med Internet Res. 2017 Nov 24;19(11):e394. doi: 10.2196/jmir.7997 (PMC5722976; doi:10.2196/jmir.7997)
Supplement: Multimedia Appendix 3 [file jmir_v19i11e394_app3.pdf]

Table S3: Results of included studies

Study	Results
Anand 2015	Of 11,120 MSM receiving online counselling, 8,288 were referred for HIV testing, 1,181 received HIV testing, 183 tested positive (HIV prevalence of 15.5%). 1,112 MSM living with HIV were advised on where and how to access HIV treatment medications.
Anand 2016	Of 97 individuals, 25 (26%) opted for eHTC and 72 (74%) for online counseling and clinic-based testing. HIV prevalence was higher among eHTC than clinic-based testing participants (16% vs 1.4%, p=0.02).
Bauermeister 2015	Intervention group (tailored) [N= 68]: 18 (26%) tested for HIV;<br>Control group (test-locator) [N= 36]: 4 (11%) tested for HIV.
Brady 2014	Of 14,208 requesting tests kits, 9,948 (70%) returned them and 118 (1%) of them tested positive.
Buzdugan 2016	Of 61 participants, [33 in Arm 1 (no incentives) and 28 in Arm 2 (with incentives)], 5 (8%) tested for HIV and syphilis.
Elliot 2012	321 visitors completed risk assessment, 209 clicked through for information on home sampling. 132 test kits were requested, 73 (55%) were returned, 4 (5.5%) of which were new HIV diagnoses.
Elliot 2016	66,579 visited website, 17,361 completed risk assessment. 10,323 (93%) ordered sample kit, 5,696 (55%) returned samples, 96 (2%) were confirmed positive including 82 new diagnoses.
Horvath 2013	There was not a significant difference in improvement for overall ART adherence in intervention (n=66) compared to control condition (n=57). But there was greater improvement in taking ART within 2 hours of scheduled dose and correctly with food.
Huang 2015	There were 16,328 unique visitors and 667 HIV self-test requests. Of the 112 survey participants, 4 reported HIV positive results after testing and sought medical care. The cost per person was $39 and cost per new case of HIV identified was $4,400.
Hyden 2016	Of 274 participants surveyed, 56% were very/somewhat likely to get tested again in next 30 days.
Jones 2015	Of 305 people engaged or clicked on Facebook survey, 9 (3%) requested HIV test at home. 5 (56%) made appointments and tested for HIV. None were detected positive.
Ko 2013	Of 1037 survey participants, those using the intervention website (n=552) were more likely to have HIV tests within 6 months (43.89% versus 22.31%, P<.001) than those using the control website (n=485).

Lampkin 2016	816 (90%) contacts with MSM resulted from Grindr, 562 (69%) remained engaged. Of those engaged, 35% received some combination of counseling, referrals, testing, treatment, and/or follow-up. 14 MSM tested for HIV and 1 new case was identified.
Mendizabal-Burastero 2016	7,244 men were recruited, nearly 50% tested for HIV. Nearly 200 HIV cases were diagnosed [22% of the estimated HIV cases in MSM in Guatemala City].
Munro 2016	Of those who asked questions about sexual health, 29.4% asked about HIV testing.
Patel 2016	Recent HIV testing from baseline to follow-up increased from 32% to 44%;(n=130, p< 0.05). Higher proportion of participants in the avoidance condition reported recent testing or intention to test (82%), compared to approach condition (65%).
Rhodes 2016	The odds of past 12-month HIV testing at posttest compared to baseline for intervention participants (n=692) was 2.9 times as high as the odds of testing at posttest compared to baseline for comparison participants (n=600).
Rhodes 2011	The intervention significantly increased self-reported HIV testing among chatters from 44.5% at pretest (n=346) to nearly 60% at post-test (n=315).
Roberts 2015	During the first six months of 2014, the number of HIV tests conducted with MSM had an increase of 29% compared with the average number of tests per quarter in 2013.
Sun 2014	Of the 2,709 interactions with app users, 240 were informational chats with health educator. 63 users requested referral for HIV testing (26.3% conversion rate).
Tang 2015	HIV test uptake was similar between the crowdsourced arm (37%,114/307) and the health marketing arm (35%, 111/317). Among those tested, 31% (69/225) reported a new HIV diagnosis.
Washington 2016	Those receiving the intervention were 7 times more likely to have tested for HIV than those in the control group at 6 weeks follow-up.
West 2016	Of 55 people booking an appointment online, 34 (62%) attended. 24% (8/34) reported never previously testing for HIV.
Young 2015	Intervention groups (N=278): 252 retained; 31% (77/252) requested HIV test and 17% (43/252) tested for HIV. Control groups (N=278): 246 retained; 15% (36/246) requested HIV test and 7% (16/246) tested for HIV

Young 2013	Intervention group (n=57): 25 [44%] requested testing kits; 9 [16%] returned it and 8 [14%] followed up to obtain test results. Control group (n=55): 11 [20%] requested testing kits; 2 [4%] returned it and none followed up to obtain test results.
Zou 2013	Among the MSM tested and completed questionnaire (n=399), 153 (36%) were through banners on gay websites and 80 (19%) from gay chat rooms. HIV testing recruitment yields through instant messaging, online gay chat room, mobile phone contact, and email were 1:4, 1:6, 1:10, and 1:140, respectively (no. not available for banners on gay websites).
